# Factors contributing to the duration of postpartum abstinence among Nigerian women: semi-parametric survival analysis

**DOI:** 10.1016/j.heliyon.2018.e01032

**Published:** 2018-12-17

**Authors:** A.F. Fagbamigbe, I.E. Awoyelu, O.L. Akinwale, T.Y. Akinwande, B.K. Enitilo, O. Bankole

**Affiliations:** a Department of Epidemiology and Medical Statistics, Faculty of Public Health, College of Medicine, University of Ibadan, Nigeria; b Centre for AIDS Research, Department of International Health, Bloomberg School of Public Health, Johns Hopkins University, Baltimore, USA

**Keywords:** Reproductive medicine, Public health

## Abstract

**Background:**

The duration of postpartum abstinence is on the decrease but has not been met with increased uptake of contraceptive in Nigeria. This imbalanced transition could result in shorter birth intervals and worsen maternal and child health outcomes. There is a paucity of information on the duration and predictors of time to end of postpartum abstinence in Nigeria. This study was aimed at understanding the time to end of postpartum abstinence and factors predicting the duration in Nigeria.

**Methods:**

The NDHS 2013 data was used. Data of all women who had ever given birth were included. The time to end of postpartum abstinence was censored among currently breastfeeding mothers. The Kaplan Meier Product Limit method was used to estimate the survival and hazard function while the Cox regression was used to fit a model for time to end of postpartum abstinence at 5% significance level. Data were weighed and provisions were made for multicollinearity.

**Results:**

The overall average duration of postpartum abstinence in Nigeria is 3 month. In all, 58% ended postpartum abstinence within the first three months while 18%, 10%, and 14% ended it within 4–6 months, 7–12 months and after one year respectively. Postpartum abstinence did not last beyond 3 months among 83% of the women in the North-West region, compared with 23% in the North Central region, and 34% in the South East. The Muslims had the highest proportion of women who ended postpartum abstinence within the first three months after delivery at 72% compared with Catholic women (31%). The median time to end of postpartum abstinence was lowest (2 months) among women from North West, Muslims, in poorest wealth quintiles and those with no education. The “hazard” of earlier resumption of sexual activity after birth was over 3 times more likely among women in the North West than those in the North Central (aHR = 3.09, 95% CI: 2.95–3.24). Women using contraceptives had a 40% hazard of ending postpartum abstinence earlier. Rural women were 7.6% times less likely to end postpartum abstinence compared to their urban counterpart. Women from rich households have an excess risk of 14% to end postpartum abstinence early compared to women from poor households.

**Conclusion:**

Women of reproductive age in the North West, who are Muslims and with no education are at higher risk of ending postpartum abstinence early. Hence, policymakers and reproductive health stakeholders should design effective intervention targeted at this group of women as a means of controlling fertility.

## 1 Background

Postpartum Abstinence is the period of voluntary sexual abstinence after pregnancy delivery and when combined with postpartum amenorrhea is the period of insusceptibility to pregnancy (National Population Commission (Nigeria) and ICF International, 2014). Globally, the period of postpartum abstinence is on the decrease but it has been met with increased prevalence of contraceptive use (World Health Organization, 2010). However, the contrary is the situation in developing countries. In Nigeria for instance, while the postpartum abstinence reduced overtime (Caldwell and Caldwell, 1981), low contraceptive use prevailed (National Population Commission (Nigeria) and ICF International, 2014; National Population Commission (Nigeria) and ICF International, 2009). The implication of this imbalanced transition is a short birth interval, and this might explain the relatively constant high fertility and maternal mortality the country has been experiencing for over a decade. The same inference can be made for the prevalent high under-five mortality in Nigeria, since short birth interval does not only affects the health of a mother but also that of the child (Ball et al., 2014; Batyra, 2016; Chen et al., 2014; Zhang et al., 2017).

Studies conducted in the 1980s (Caldwell and Caldwell, 1981; van de Walle and van de Walle, 1989) showed that Nigeria and many other African countries had a postpartum abstinence period ranging from 12 to 15 months. A recent qualitative study (Mbekenga et al., 2013) explained the reason for such interval to be as a result of cultural beliefs that sperm can spoil the breast milk and thus affect the health of the child (van de Walle and van de Walle, 1991). However, more recent studies (National Bureau of Statistics Tanzania and ICF - Macro, 2011; National Population Commission (Nigeria) and ICF International, 2014) have shown a reduced length of postpartum abstinence (3 months) in some African countries. This might partially explain the reason most African countries, Nigeria included, are experiencing a relatively constant high fertility for decades. This shorter length of postpartum abstinence could significantly reduce the positive influence which education, improved socio-economic status and increased female decision making power have on fertility.

As much as longer postpartum abstinence is desirable in a country with low contraceptive prevalence, the advent of HIV/AIDS and other STIs in countries like Nigeria decreases the need for its promotion. An earlier study in South West Nigeria reported that men result to extramarital sex during wife postpartum abstinence period (Lawoyin and Larsen, 2002) which exposes men to STDs and by extension the, the wives. Then, is shorter postpartum abstinence preferable in order to protect the women from contracting STIs from her spouse but at the risk of having short birth intervals which are detrimental to her health and that of her child? (Cleland et al., 1999).

Postpartum abstinence is one of the factors determining fertility, maternal and child mortality, meanwhile very few research works have been published with respect to postpartum abstinence in Nigeria in recent time except the few regional and relatively old studies of postpartum abstinence (Sule-Odu et al., 2008; van de Walle and van de Walle, 1989). Caldwell's work on postpartum abstinence in Nigeria was done 30 years ago and might not represent its current situation in the country. The need to re-assess the Nigeria situation and update the body of knowledge as far as postpartum abstinence is concerned with regards to the time to resumption of sexual intercourse after birth and factors that could influence such timings motivated this study. A good knowledge of the timings as well as an understanding of its prognostic factors will enable policymakers and other sexual and reproductive health stakeholders to make more informed decisions on family planning and contraception programme including reproductive health education to high-risk sub-populations in Nigeria.

The current study is poised at providing answers to “what is the timing of the end of postpartum abstinence in Nigeria?”, “what are the factors affecting the time to end of postpartum abstinence among women of reproductive ages?”; “Does contraceptive use affect the timing of postpartum sexual intercourse resumption?”, “does mode of delivery affect the timing of the end of postpartum abstinence?” We, therefore, set to determine the timing of postpartum abstinence among women of reproductive age in Nigeria, assess the effect of women characteristics on the timings, and to determine the influence of delivery mode and contraceptive use on the timing of postpartum sexual intercourse resumption.

## 2 Materials and methods

A nationally representative data from the Nigeria Demographic and Household Survey (NDHS) 2013, (National Population Commission (Nigeria) and ICF International., 2014) was used in this study. The survey was designed to provide information on population and health indicator estimates at the national, zonal and state level. It used enumeration areas (EA) that were prepared from the 2006 population census of Nigeria as primary sampling units. A stratified three-stage cluster design was used to select a nationally representative sample. The dataset had a detailed reproductive history of the women interviewed.

### 2.1 Data

We extracted birth and sexual activity history of all women who have had at least one childbirth either currently breastfeeding or had stopped. Information was limited to the most recent births for women who had had more than one birth during the period. Women who were not sure of the time they resumed sexual activity after their last birth were excluded in the study. While the time to end of postpartum abstinence of women who had already ended postpartum abstinence was used, the time spent so far on postpartum abstinence among currently breastfeeding women was censored.

### 2.2 Variables

The outcome variable in this study is the duration of postpartum abstinence, which is time to resumption of postpartum sexual intercourse. The explanatory variables include marital status (since pregnancy and postpartum sexual intercourse in some instances are independent of marital status); respondents' region, age, religion, use of contraceptive, place of residence, wealth status, family type, woman's education, partner's education, mode of delivery, age difference between woman and partner, total child ever born, current working status of respondent, breastfeeding practice, and duration of postpartum amenorrhea. The choice of these variables is based on earlier studies (Adhikari and Podhisita, 2010; Fatusi and Blum, 2008; Kennedy and Visness, 1992) which have shown that socio-demographic factors could explain sexual behaviours and fertility levels as well as postpartum abstinence. Other factors like mode of delivery (Alum et al., 2015) have also been linked with postpartum abstinence. Due to available screening questions that determine whether exclusive breastfeeding was practiced or not, the number of participants that could be assessed was only 2930 compared to 31828 participants that had responses on other women's characteristics considered.

### 2.3 Data analysis

We used both descriptive statistics and survival analysis techniques to analyze the data. In the descriptive analysis, postpartum abstinence was grouped into four; 0–3 months, 4–6 months, 7–12 months and after 12 months. Literature is scarce on standard period to be regarded as early or late postpartum abstinence. However, sexual intercourse can resume in the second month when perinea healing must have taken place. Using a conservative approach, we used this as a benchmark to collapse postpartum abstinence period into short, moderate, long and very long for 0–3 months, 4–6 months, 7–12 months and after 1 year respectively. Although the variable “breastfeeding practice” was significant at the bivariate level, it was not included in the multiple Cox regression analysis due to a very low number of respondents compared with other explanatory variables. Its inclusion will distort the outcomes as analysis will be restricted to only 2930 respondents ratger than over 30000.

### 2.4 The rationale for use of survival analysis

Survival analysis was adopted in the analysis of this study because of the nature of our dependent variable, time to end of postpartum abstinence, which is a time variable. Excluding women who are currently on postpartum abstinence from analysis could seriously bias the findings. The Kaplan Meier Product Limit method was used to estimate the survival and hazard function due to its ability to take censored observation into consideration.

For those who had resumed sex after childbirth, the survival time is the time period they waited after childbirth before resuming sexual intercourse. However, not all women who were studied had ended postpartum abstinence as at the time of the survey. The duration of postpartum abstinence of these sets of women were right censored as of the survey date and were included in the analysis. They constituted about 11% of the eligible respondents. The survival time of this category of women was computed as the number of months between last childbirth and the survey date. The survival function S(t) – (Eq. (1)), is the probability that a woman will continue postpartum abstinence after a particular time “t” was measured in months was estimated and plotted. In this study, the S(t) meaning the probability that. The hazard ratio, h(t) is the instantaneous failure rate at which a woman who had not resumed postpartum sexual intercourse by time “t” will resume sexual intercourse immediately after time “t”.

(1) $$S\left(t\right)=\;\overset{k}{\underset{j=1}{\prod }}\frac{\left(n_{j}-d_{j}\right)}{n_{j}}$$

Where n_j_ is the number of subjects observed at time t_j_ and d_j_ is the number of subjects that have ended postpartum abstinence at time t_j_. The Log-rank test was used to compare the survival experience between different categories of the characteristics studied. We estimated the Incidence Rate (IR), which is the probability that a woman will resume sexual activity after a childbirth per unit time (month).

Furthermore, the survival time was modelled with respect to certain prognostic factors using the Cox proportional hazard regression. The coefficients in the Cox regression model indicates the changes expected in the duration of postpartum abstinence in one category of a variable compared with another category. The hazard ratio (HR), expressed as the exponentials of the coefficients, indicates a higher likelihood of exposure to event of interest if HR > 1, HR < 1 implies lower exposure; while HR = 1 suggest no significant difference in exposure. Variables significant in the independent Cox regression were used in the multiple Cox regression while controlling for confounders.

We made provision for intracluster correlation and also weighed the data to adjust for differences in population sizes of each state in Nigeria and the FCT. Statistical significance was determined at 5%. Stata (version 14) was for all the data analysis.

### 2.5 Ethical consideration

The National Health Research Ethics Committee reviewed the NDHS proposal and approved the research work with reference number: NHREC/01/01/2007 (National Population Commission (Nigeria) and ICF International, 2014).

## 3 Result

In all, 89% of the eligible women have ended postpartum abstinence as of the time of the survey (Not shown in the Tables). The overall average duration of postpartum abstinence in Nigeria is 3 months as shown in Table 1. In all, 58% ended postpartum abstinence within the first three months while 18%, 10%, and 14% ended it within 4–6 months, 7–12 months and after one year respectively. Postpartum abstinence did not last beyond 3 months among 83% of the women in the North-West region, compared with 23% in the North Central region, 34% in the South East, 38% in the South-South and 39% in the South West region ended their postpartum abstinence within the first three month after delivery.

**Table 1:** Distribution of respondents by socio-demographic characteristics, duration of postpartum abstinence and median month to end of postpartum abstinence.

Characteristics	%	Median Time to end of PA	Postpartum Abstinence (Months)			
			0–3	4–6	7–12	>1 year
Region						
North Central	13.6	8	23.1	20.5	24.8	31.6
North East	17.5	3	67.7	13.6	8.0	10.7
North West	37.0	2	83.2	10.8	1.3	4.7
South East	8.9	6	33.6	26.7	15.0	24.8
South South	9.2	4	38.3	26.0	16.4	14.8
South West	13.7	5	38.8	26.0	16.4	18.8
Age in Group						
15–19	5.0	3	58.0	13.8	7.3	20.8
20–24	19.6	3	59.9	16.9	9.2	14.0
25–29	27.9	3	58.9	18.9	9.4	12.8
30–39	37.4	3	56.4	18.9	9.6	13.4
40–49	10.1	3	56.4	16.6	11.7	15.3
Religion						
Catholic	8.6	6	30.9	25.5	20.7	22.9
Other Christian	28.3	5	36.5	27.2	16.1	20.1
Islam	62.3	2	71.6	12.8	5.8	9.8
Traditional	0.9	4	46.2	16.1	17.9	19.7
Contraceptive Use						
Do not use	84.8	3	59.8	15.9	9.0	15.3
Using a method	15.2	4	47.4	29.8	16.2	6.6
Place of residence						
Urban	35.0	3	53.8	22.3	10.0	13.9
Rural	65.0	3	60.1	15.8	10.2	14.0
Wealth Status						
Poorest	46.7	2	68.4	12.1	7.4	12.1
Middle Class	18.9	4	45.7	20.1	14.7	19.6
Richest	34.5	4	50.3	25.0	11.3	13.4
Family Type						
Monogamous	67.5	3	56.3	20.1	10.7	12.9
Polygamous	32.5	3	65.6	13.9	8.4	12.1
Education						
No Education	49.2	2	72.0	11.9	5.9	10.2
Primary	19.3	4	44.0	20.4	15.8	19.8
Secondary	25.8	4	42.9	26.1	13.8	17.2
Tertiary	5.8	3	51.8	26.3	10.2	11.6
Partner Education						
No Education	39.8	2	74.2	11.2	5.1	9.4
Primary	19.0	4	48.5	20.1	13.2	18.1
Secondary	29.1	4	46.2	24.6	14.0	15.1
Tertiary	12.1	3	53.2	21.7	11.3	13.8
Caesarean delivery						
No	97.9	3	58.3	17.8	10.0	13.8
Yes	2.1	5	35.8	28.9	15.9	19.4
Age Difference with Partner						
Partner Younger	0.9	4	56.7	19.4	13.1	10.8
0–2 years	6.6	4	46.4	23.5	13.4	16.7
3–5 years	20.0	3	53.2	20.6	11.6	14.6
Above 5 years	72.5	3	62.2	16.9	9.1	11.8
Children Ever Born						
1–2 Children	28.8	4	51.9	20.2	10.6	17.3
3–4 Children	30.8	3	57.9	19.7	10.4	12.0
Above 4 children	40.4	3	62.1	15.2	9.6	13.1
Current Work Status						
Not Working	31.0	3	66.3	14.7	6.4	12.5
Working	69.0	3	54.1	19.5	11.8	14.6
Postpartum Amenorrhea						
0–3 months	28.8	3	55.4	16.5	7.3	20.8
4–6 months	19.0	3	52.3	25.5	9.3	13.0
7–12 months	28.7	3	55.1	18.6	15.4	10.9
>1 year	23.6	3	67.0	14.0	7.8	11.1
Breastfeeding Practice*						
Not Practicing EBF	82.5	-	44.8	3.4	0.7	51.1
Practice EBF	17.5	-	28.6	1.8	0.0	69.6
**Total**	**31828**	3	57.9	18.0	10.1	14.0

PA, postpartum abstinence; EBF, exclusive breastfeeding.

*Only 2930 respondents.

The Muslims had the highest proportion of women who ended postpartum abstinence within the first three months after delivery at 72% compared with Catholic women (31%) and 37% among other Christian women and only 19% of the traditionalist. Nearly a quarter (23%) of the Catholics continued postpartum abstinence after a year since delivery. More than half (60%) of the women who do not use contraceptives ended postpartum abstinence within the first three months, compared with nearly half (47%) among women using contraceptives.

About two-thirds of women from households in the poorest wealth quintiles ended postpartum abstinence during the first three months of child delivery compared with about half of those from the middle (46%) and richest (50%) wealth quintiles. Two-third of the unemployed women resumed sexual activity within three months after birth compared to the working class where 54%ended postpartum abstinence within the same period. About three-quarter (72%) of the uneducated women ended postpartum abstinence within the first three months with a median time to resumption of sexual intercourse after delivery of 2 months. Among women who had their delivery without caesarean section, 58% ended postpartum abstinence within the first three months compared to about one-third (36%) of women who delivered through the caesarean section as shown in Table 1.

The median time to end of postpartum abstinence was lowest (2 months) among women from North West, Muslims, in poorest wealth quintiles and those with no education and highest among those from the North Central (8 months), been a Catholic (6 months) been a Christian (5 months) and delivered last child through caesarean operation (5 months).

In Table 2, we present the incidence rate, the median survival time and the outcome of log-rank test used to determine the significance of differences in the duration of postpartum abstinence among the different categories of the explanatory variables. The incidence rate was 0.92 in the North West compared to 0.37 in the North Central. The incidence rate was also higher among women practicing Islam than the Catholics (0.80 vs 0.47). Similarly, the incidence rates were significantly higher among those in polygamy, with no education, in poorest wealth quintiles, delivery through caesarean operation, with wider age difference between spouses and living in the rural areas. The median survival time also followed similar patterns. With respect to region of residence, it was observed that North-Central experienced the longest postpartum abstinence with a median survival time of 2 months while North-West experienced the shortest with a median survival time of 8 months. The median survival time for all age groups in this study was 3 months. However, the Log-rank test as shown in Table 2, shows that there exists a significant difference in survival distribution of time to end of post-partum abstinence by age group. The Log-rank test shows that all explanatory variables considered in this study had a significantly different survival distribution except the variable “age difference with partner”.

**Table 2:** The Incidence rate, median survival time and log-rank test, by respondents' characteristics.

Variable	Incidence rate	Median survival time to end of PA	Log-rank test
Region			
North Central	0.37	8 (7.7–8.3)	
North East	0.74	3 (2.9–3.1)	<0.001
North West	0.92	2 (1.9–2.0)	<0.001
South East	0.48	6 (5.8–6.2)	<0.001
South South	0.63	4 (3.9–4.1)	<0.001
South West	0.51	5 (4.8–5.2)	<0.001
Age in Group			
15–19	0.65	3 (2.9–3.1)	
20–24	0.69	3 (2.9–3.1)	<0.001
25–29	0.69	3 (2.9–3.1)	<0.001
30–39	0.67	3 (2.9–3.1)	<0.001
40–49	0.65	3 (2.8–3.2)	<0.001
Religion			
Catholic	0.47	6 (5.7–6.2)	
Other Christian	0.53	5 (4.9–5.1)	<0.001
Islam	0.80	2 (1.9–2.0)	<0.001
Traditional	0.53	4 (2.8–5.2)	0.04
Contraceptive Use			
Do not use	0.69	3 (2.9–3.0)	
Using a method	0.64	4 (3.9–4.1)	<0.001
Place of residence			
Urban	0.66	3 (2.9–3.1)	
Rural	0.70	3 (2.9–3.0)	<0.001
Wealth Status			
Poorest	0.76	2 (1.9–2.0)	
Middle Class	0.56	4 (3.8–4.2)	<0.001
Richest	0.66	4 (3.9–4.1)	<0.001
Family Type			
Monogamous	0.68	3 (2.9–3.1)	
Polygamous	0.74	3 (2.9–3.0)	<0.001
Education			
No Education	0.80	2 (1.9–2.0)	
Primary	0.56	4 (3.8–4.2)	<0.001
Secondary	0.57	4 (3.9–4.1)	<0.001
Tertiary	0.67	3 (2.9–3.1)	<0.001
Partner Education			
No Education	0.82	2 (1.9–2.0)	
Primary	0.60	4 (3.9–4.1)	<0.001
Secondary	0.61	4 (3.9–4.1)	<0.001
Tertiary	0.66	3 (2.9–3.1)	<0.001
Caesarean delivery			
No	0.69	3 (2.9–3.0)	
Yes	0.52	5 (4.6–5.4)	<0.001
Age Difference with Partner			
Partner Younger	0.65	4 (3.6–4.4)	
0–2 years	0.62	4 (3.8–4.2)	0.12
3–5 years	0.65	3 (2.9–3.1)	0.72
Above 5 years	0.72	3 (3.0–3.1)	0.06
Children Ever Born			
1–2 Children	0.63	4 (3.9–4.1)	
3–4 Children	0.69	3 (2.9–3.1)	<0.001
Over 4 children	0.71	3 (2.9–3.0)	<0.001
Current Work Status			
Not Working	0.75	3 (2.9–3.0)	
Working	0.65	3 (2.9–3.0)	<0.001
Postpartum Amenorrhea			
0–3 months	0.55	3 (2.9–3.1)	<0.001
4–6 months	0.52	3 (2.9–3.1)	<0.001
7–12 months	0.55	3 (2.9–3.1)	<0.001
>1 year	0.67	3 (2.9–3.1)	<0.001
Breastfeeding Practice			
Not Practicing EBF	0.44	3 (2.8–3.2)	<0.001
Practice EBF	0.28	3 (2.8–3.1)	<0.001

PA postpartum abstinence.

**Fig. 1:**
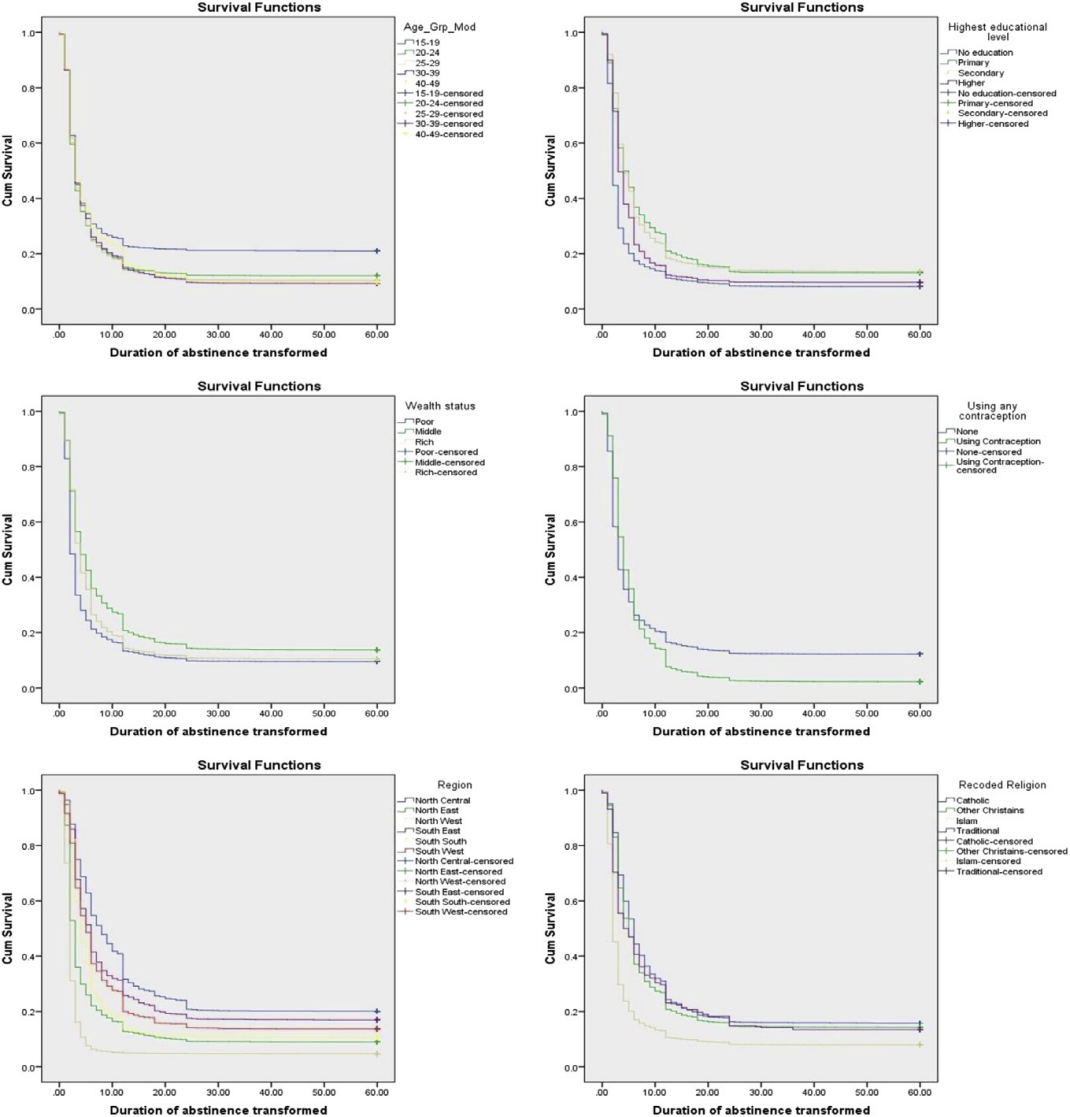
Survival functions of time to end of postpartum abstinence among women of reproductive age in Nigeria by selected characteristics.

Fig. 1 shows the survival curves for the distribution of time to end of postpartum abstinence by selected respondents characteristics. The curves showed that the survival experience across the different categories of their characteristics is distinctly different.

In Table 3, the outcome of the Cox proportional hazard model is presented. The hazard of an end to postpartum abstinence among women from the North-West was 3.18 times higher than among women from the North-Central (HR = 3.18 95% CI: 3.05–3.31). The risk of resuming sexual intercourse after childbirth is in excess of 92% for women in the North-East compared to those in the North-Central (HR = 1.92, 945% CI:1.83–1.99). Women in virtually all other age groups were about 20% more likely to end postpartum abstinence than women aged 15–19 years. With respect to religion, Muslim women compared to Catholic women had a higher hazard of ending postpartum abstinence (HR = 1.83, 95% CI: 1.75–1.91). Similarly, women from richer households were about 20% less likely to end postpartum abstinence compared with women from poorer households (HR = 0.81, 95% CI: 0.79–0.83) as shown in Table 3. The hazard of ending postpartum abstinence was also significantly higher among women in polygamy, with no education, using contraceptives, residing in rural areas, whose spouse had no education, delivery through caesarean operation, of similar age with spouse, with fewer children, currently unemployed, having longer duration of postpartum amenorrhea and not exclusively breastfeeding child in the first six month after delivery.

**Table 3:** The unadjusted determinants of time to end of postpartum abstinence among women of reproductive age in Nigeria.

Variable	Categories	HR	(95% CI)
Region	North Central	Reference	
	North East	1.92	(1.83–1.99)*
	North West	3.18	(3.05–3.31)*
	South East	1.17	(1.10–1.23)*
	South South	1.49	(1.41–1.55)*
	South West	1.30	(1.23–1.36)*
Age in Group	15–19	Reference	
	20–24	1.22	(1.15–1.30)*
	25–29	1.25	(1.17–1.33)*
	30–34	1.25	(1.17–1.33)*
	35–39	1.23	(1.15–1.31)*
	40–49	1.18	(1.11–1.27)*
Religion	Catholic	Reference	
	Other Christian	1.08	(1.03–1.13)*
	Islam	1.83	(1.75–1.91)*
	Traditional	1.13	(0.992–1.28)
Contraceptive Use	Do not use	Reference	
	Using a method	1.05	(1.02–1.09)*
Place of residence	Urban	Reference	
	Rural	1.06	(1.04–1.09)*
Wealth Status	Poor	Reference	
	Middle Class	0.71	(.691 - .737)*
	Rich	0.81	(.787 - .830)*
Family Type	Monogamous	Reference	
	Polygamous	1.13	(1.10–1.16)*
Education	No Education	Reference	
	Primary	0.65	(0.63–0.67)*
	Secondary	0.64	(0.62–0.66)*
	Tertiary	0.77	(0.73–0.81)*
Spouse Education	No Education	Reference	
	Primary	0.66	(0.64–0.68)*
	Secondary	0.66	(0.64–0.68)*
	Tertiary	0.73	(0.70–0.76)*
Delivery through CS	No	Reference	
	Yes	0.77	(0.70–0.83)*
Age Difference with Partner	Spouse Younger	Reference	
	0–2 Years Difference	0.92	(0.81–1.04)
	3–5 Years Difference	0.98	(0.86–1.11)
	Above 5 Years	1.11	(0.99–1.26)
Child Ever Born	1–2 Children	Reference	
	3–4 Children	1.20	(1.16–1.24)*
	Above 4 Children	1.25	(1.21–1.28)*
Current Work Status	Not Working	Reference	
	Working	0.89	(0.87–0.91)*
Postpartum Amenorrhea	0–3 months	Reference	
	4–6 months	1.16	(1.11–1.20)*
	7–12 months	1.24	(1.20–1.28)*
	>1 year	1.45	(1.40–1.50)*
Breastfeeding Practice	Practice EBF	Reference	
	Not Practicing EBF	1.96	(1.65–2.34)*

*Significant at 5% HR Hazard Ratio.

After controlling for other variables, women in the North West had the highest likelihood of ending postpartum abstinence. They were 215% more likely than women in the North Central region to resume sexual activity earlier after birth (aHR = 3.15, 95% CI: 3.00–3.30). Those in the South East were 34% times more likely to end postpartum abstinence compared with women in the North Central. The chances that women aged 25–29 years and women aged 30 and 34 years will stop the postpartum abstinence were 23% and 19% times higher than among women aged 15–19 years.

The adjusted hazard ratio (aHR) of 1.40 was found among women using contraceptives compared with those that did not use to end postpartum abstinence. Table 4 also shows that rural women were 7% less likely to end postpartum abstinence compared to their urban counterparts. With respect to wealth status, the women from households in richer wealth quintiles have an excess risk of 14% of ending postpartum abstinence compared to women from households in poorer wealth quintiles. Women, who were delivered of their baby through caesarean section, were 13% times less likely to resume sexual intercourse as women who had a normal child delivery. Other significant characteristics are the numbers of children ever born, duration of postpartum amenorrhea and spouse and women educational attainments as shown in Table 4.

**Table 4:** The adjusted determinants of time to end of postpartum abstinence among women of reproductive age in Nigeria.

Variable	Categories	aHR	(95% CI)
Region	North Central	Reference	
	North East	2.00	(1.91–2.09)*
	North West	3.15	(3.00–3.30)*
	South East	1.34	(1.26–1.43)*
	South South	1.73	(1.63–1.83)*
	South West	1.21	(1.15–1.28)*
Age in Group	15–19	Reference	
	20–24	1.22	(1.14–1.30)*
	25–29	1.23	(1.15–1.32)*
	30–34	1.19	(1.10–1.28)*
	35–39	1.10	(1.01–1.20)*
Religion	Catholic	Reference	
	Other Christian	1.04	(0.99–1.10)
	Islam	1.28	(1.21–1.36)*
	Traditional	0.99	(0.87–1.15)
Contraceptive Use	Do not use	Reference	
	Using a method	1.40	(1.35–1.47)*
Place of residence	Urban	Reference	
	Rural	0.93	(0.90–0.96*
Wealth Status	Poor	Reference	
	Middle Class	0.97	(0.94–1.01)
	Rich	1.14	(1.09–1.19)*
Family Type	Monogamous	Reference	
	Polygamous	0.97	(0.94–0.99)*
Education	No Education	Reference	
	Primary	0.96	(0.92–0.99)*
	Secondary	0.98	(0.95–1.05)
	Tertiary	1.19	(1.11–1.28)*
Spouse Education	No Education	Reference	
	Primary	0.89	(0.85–0.92)*
	Secondary	0.91	(0.87–0.95)*
	Tertiary	0.91	(0.87–0.97)*
Delivery through CS	No	Reference	
	Yes	0.87	(0.79–0.95)*
Child Ever Born	1–2 Children	Reference	
	3–4 Children	1.11	(1.07–1.15)*
	Above 4 Children	1.08	(1.03–1.13)*
Current Work Status	Not Working	Reference	
	Working	1.01	(0.98–1.03)
Postpartum Amenorrhea	0–3 months	Reference	
	4–6 months	1.24	(1.19–1.29)*
	7–12 months	1.31	(1.31–1.41)*
	>1 year	1.36	(1.36–1.46)*

*Significant at 5% aHR adjusted hazard ratio Breastfeeding practice dropped due to low sample size.

## 4 Discussion

The current study explored the time to end of postpartum abstinence. We included the time already spent in postpartum abstinence among women who were currently breastfeeding. In all, we found postpartum abstinence to have ended after three months among the women studied and this varied by their characteristics. Respondents' region, age, religion, use of contraceptive, place of residence, wealth status, family type, woman's education, partner's education, mode of delivery, and total child ever born were found to be significantly associated with duration of postpartum abstinence. The postpartum sexual behaviour of Nigerian women seemed to be experiencing a transition. This is in light of the fact that studies carried out towards the end of the 20^th^ century (Caldwell and Caldwell, 1981; van de Walle and van de Walle, 1989) reported longer postpartum abstinence among women from various part of Africa while more recent study (National Bureau of Statistics Tanzania and ICF - Macro, 2011) found a much shorter postpartum abstinence period which corroborated our findings. The long postpartum abstinence observed in the late 20^th^ century in many parts of Africa was influenced by cultural beliefs. Some investigators (van de Walle and van de Walle, 1991) noted that the belief that sperm might be poisonous to the breast milk was one of the major reasons for a lengthy postpartum abstinence period. However, with an increase in reproductive health knowledge, the influence of such cultural beliefs might have reduced and could serve as an explanation for the prevailing reduced postpartum abstinence period in most African countries including Nigeria.

Compared to the Caldwell Study (Caldwell and Caldwell, 1981) which reported that postpartum abstinence among women not using a contraceptive method was 15 months, we discovered that the median length of postpartum abstinence among such women is only 3 month. This shows that there has been a great decline in the length of postpartum abstinence from 1970 to date. However, the postpartum abstinence of average of three months found in the current study is lower than the 4.5 months reported in a Southwestern Nigerian study (Sule-Odu et al., 2008). There appears to be a consistent decline in postpartum abstinence duration in Nigeria.

Although reduced postpartum abstinence can be beneficial since it reduces the risk of STI transmission as studies (Achana et al., 2010; Cleland et al., 1999; Lawoyin and Larsen, 2002) have shown that spouses of abstaining wife are likely to engage in unsafe extramarital sexual activities. However, the consequences of such reduction are equally threatening. The devastating effect of reduced postpartum abstinence could be more severe in countries like Nigeria where contraceptive prevalence is low (Fagbamigbe et al., 2018; National Population Commission (Nigeria) and ICF International, 2014) and abortion is prohibited (Oye-Adeniran et al., 2004). Most women resume sexual activity at an earlier time than what it used to be without the use of contraceptives; exposing them to the risk of unwanted pregnancy. A woman who eventually fall a victim of unwanted pregnancy are left with two devastating options. One option is to leave the pregnancy and risk both her health as a result of birth in quick succession and the health of the child recently given birth to as a result of the reduced care due to pregnancy (Setty-Venugopal and Upadhyay, 2002). The second option will be to go for an unsafe abortion, which poses an increased risk of maternal mortality (Haddad and Nour, 2009).

The multiple Cox regression analysis revealed a lower adjusted hazard ratio among teenage mothers. This suggests that younger women are more likely to extend postpartum abstinence than women of other reproductive ages. This is quite intuitive as teenage mothers are more likely to be out of wedlock compared to older age group; hence they are faced with a lower pressure for sexual intercourse after birth. This finding is similar to an earlier finding (Sule-Odu et al., 2008).

Women from other regions in Nigeria have a higher likelihood of earlier end of postpartum abstinence than women in North-Central Nigeria. Women in the North-West have the highest likelihood of an earlier end to postpartum abstinence compared with women in the North-Central region. Although most women in the North West and North-Central practices Islam (Fagbamigbe et al., 2015), they are of different culture and traditional practices. This might have informed the wide disparity found in their likelihood to end postpartum abstinence. It might be necessary to conduct further studies to elicit the reason for the long postpartum abstinence period in the north central region compared to other regions of Nigeria.

A study on the role of religion in developing countries, employing data from the DHS of 30 countries, noted that fertility among Muslim is significantly higher than among the Catholics while very little difference was identified in the fertility levels of Protestant Christians and Catholics (Heaton, 2011). Haeton's findings seem to correlate with the result in our study which showed that the likelihood that other Christians had a higher chance to end postpartum abstinence early compared to Catholic women wasn't significant, but Muslim women had a higher risk of ending postpartum abstinence earlier than the Catholics. With regards to contraceptive use, it was not surprising to discover that those using a contraceptive method were 40% times more likely to resume postpartum sexual intercourse at an earlier time than those not using any method. Contraceptive users might be more confident not to get pregnant soon after childbirth than women not using any method. However, the median period of postpartum abstinence end was only a month different among the users and non-users. This suggests that consequences of non-use of contraceptive were not rolled out by a longer postpartum abstinence. Therefore, considering the fact that uptake of contraceptive during postpartum is low in Nigeria (Akinlo et al., 2013), there is an urgent need to increase awareness of contraceptive use especially among postpartum women who are at risk of pregnancies in quick succession. Prenatal and postpartum contraceptive counseling can significantly increase uptake during postpartum (Zapata et al., 2015).

In this study, we also identified that higher wealth status has an association with earlier end of postpartum abstinence. Women from households in richer wealth quintile were 11% times more likely to end postpartum abstinence earlier compared to women from poorer households. Another factor which determined the time to end of postpartum abstinence was women and partner's education. Our study agrees with a qualitative study which was conducted in Burkina-Faso (Rossier and Hellen, 2014) that educated women tend to end postpartum abstinence early compared to the less educated. This gives an indication that improved female social status might reduce postpartum abstinence. There is a need to carry out qualitative studies in order to understand why women of higher social status will prefer early sexual activity after birth. However, the outcome is contrary when the respondents' partners are educated. Women who had a partner with lower education have a higher hazard of ending postpartum abstinence early.

We also found that women who were delivered of their last birth through a caesarean operation had a higher likelihood of extending postpartum abstinence. The same is the situation of women with 1–2 births and those from a polygamous family. These situations could be linked to the time needed for women to get strong for sexual activities and availability of other “sexual partners” at the disposal of the men. Lastly, our study showed that the longer the duration of postpartum amenorrhea, the higher the likelihood that a woman will delay the resumption of sexual activity. Studies are scarce on the relationship between postpartum amenorrhea and postpartum abstinence, but a possible explanation for this type of relationship could be that women who have resumed menses might have the feeling that their body has recovered from child delivery and ready for sexual activities. Qualitative studies need to be carried out to validate this assertion.

### 4.1 Conclusion

The median duration of postpartum abstinence has decreased from 15 months to 3 months between 1980 and 2013 without a significant increase in modern contraceptive uptake. Women of reproductive age in the North West, Muslim women, and women living in urban areas including those who did not have their last birth through a caesarean operation and those from monogamous family are generally at higher risk of earlier end of postpartum abstinence. Hence, Policymakers and reproductive health stakeholders should pay closer attention to women in this group. Contraceptive programmes should also be integrated with post-natal interventions in order to achieve an increased prevalence of contraceptive uptake during the postpartum period.

### 4.2 Strength and limitation of the study

The large nationally representative data used in this study has made our findings reliable. Like virtually all retrospective study, there is a potential for recall bias. This challenges would be more apparent among women who had their last birth about 4 or 5 years before the survey and were asked to recall the month they resumed sexual intercourse after last birth.

## Declarations

### Author contribution statement

A. F. Fagbamigbe: Conceived and designed the experiments; Performed the experiments; Analyzed and interpreted the data; Contributed reagents, materials, analysis tools or data; Wrote the paper.

I. E. Awoyelu, O. L. Akinwale, T. Y. Akinwande, B. K. Enitilo, O. Bankole: Performed the experiments; Analyzed and interpreted the data; Contributed reagents, materials, analysis tools or data; Wrote the paper.

### Funding statement

This research did not receive any specific grant from funding agencies in the public, commercial, or not-for-profit sectors.

### Competing interest statement

The authors declare no conflict of interest.

### Additional information

No additional information is available for this paper.
